# Inhibitory effect of *Curcuma purpurascens* BI. rhizome on HT-29 colon cancer cells through mitochondrial-dependent apoptosis pathway

**DOI:** 10.1186/s12906-015-0534-6

**Published:** 2015-02-05

**Authors:** Elham Rouhollahi, Soheil Zorofchian Moghadamtousi, Mohammadjavad Paydar, Mehran Fadaeinasab, Maryam Zahedifard, Maryam Hajrezaie, Omer Abdalla Ahmed Hamdi, Chung Yeng Looi, Mahmood Ameen Abdulla, Khalijah Awang, Zahurin Mohamed

**Affiliations:** Department of Pharmacology, Faculty of Medicine, University of Malaya, 50603 Kuala Lumpur, Malaysia; Institute of Biological Sciences, Faculty of Science, University of Malaya, 50603 Kuala Lumpur, Malaysia; Department of Chemistry, Faculty of Science, University of Malaya, 50603 Kuala Lumpur, Malaysia; Department of Biomedical Science, Faculty of Medicine, University of Malaya, 50603 Kuala Lumpur, Malaysia

**Keywords:** *Curcuma purpurascens*, Zingiberaceae, Tumerone, Colon cancer, Apoptosis, Bax/Bcl2

## Abstract

**Background:**

*Curcuma purpurascens* BI. (Zingiberaceae) commonly known as ‘Koneng Tinggang’ and ‘Temu Tis’ is a Javanese medicinal plant which has been used for numerous ailments and diseases in rural Javanese communities. In the present study, the apoptogenic activity of dichloromethane extract of *Curcuma purpurascens* BI. rhizome (DECPR) was investigated against HT-29 human colon cancer cells.

**Methods:**

Acute toxicity study of DECPR was performed in *Sprague–Dawley* rats. Compounds of DECPR were analyzed by the gas chromatography–mass spectrometry–time of flight (GC-MS-TOF) analysis. Cytotoxic effect of DECPR on HT-29 cells was analyzed by MTT and lactate dehydrogenase (LDH) assays. Effects of DECPR on reactive oxygen species (ROS) formation and mitochondrial-initiated events were investigated using a high content screening system. The activities of the caspases were also measured using a fluorometric assay. The quantitative PCR analysis was carried out to examine the gene expression of Bax, Bcl-2 and Bcl-xl proteins.

**Results:**

The *in vivo* acute toxicity study of DECPR on rats showed the safety of this extract at the highest dose of 5 g/kg. The GC-MS-TOF analysis of DECPR detected turmerone as the major compound in dichloromethane extract. IC_50_ value of DECPR towards HT-29 cells after 24 h treatment was found to be 7.79 ± 0.54 μg/mL. In addition, DECPR induced LDH release and ROS generation in HT-29 cells through a mechanism involving nuclear fragmentation and cytoskeletal rearrangement. The mitochondrial-initiated events, including collapse in mitochondrial membrane potential and cytochrome *c* leakage was also triggered by DECPR treatment. Initiator caspase-9 and executioner caspase-3 was dose-dependently activated by DECPR. The quantitative PCR analysis on the mRNA expression of Bcl-2 family of proteins showed a significant up-regulation of Bax associated with down-regulation in Bcl-2 and Bcl-xl mRNA expression.

**Conclusions:**

The findings presented in the current study showed that DECP suppressed the proliferation of HT-29 colon cancer cells and triggered the induction of apoptosis through mitochondrial-dependent pathway.

**Electronic supplementary material:**

The online version of this article (doi:10.1186/s12906-015-0534-6) contains supplementary material, which is available to authorized users.

## Background

In 1972, Kerr, Wyllie and Currie officially coined the term “apoptosis” [1-3] and the nematode of *Caenorhabditis elegans* was researched for the understanding of the mechanisms of apoptosis process involved in the mammalian cells [4,5]. Roughly 10 billion cells die through apoptosis daily in the adult human body indicating the importance of this process for maintaining homeostasis of the body [6]. Any perturbation in the regulation of apoptosis, cell survival and cell proliferation can lead to the progression of cancer cells [7]. Therefore, investigation for novel chemotherapeutic agents capable of inducing apoptosis specifically in cancer cells has been extensively carried out in order to effect cancer treatment along with a reduction in the side effects of anticancer agents [8].

Apoptosis can be initiated by a variety of intrinsic signals, such as hormones, reactive oxygen species (ROS), hypoxia, toxins and radiations, or extrinsic signals during normal development [9]. Extensive production of ROS by natural products is an important stimulus, which causes perturbation in the inner mitochondrial membrane leading to an opening of the mitochondrial permeability transition pores (MPTP) [10], collapse in mitochondrial membrane potential (MMP) [11] and leakage of several apoptogenic factors [12]. Cytochrome *c* release from mitochondria to cytosol is associated with the critical actions of Bcl-2 family of proteins in activating the caspase cascade through apoptosome formation [13]. The activation of executioner caspases induces the irreversible programmed cell death of apoptosis, which provides an effective approach to cancer therapy [5,13].

The majority of pharmaceutical progresses rely upon nutraceuticals for development of new phytochemical agents with therapeutic values [14]. *Curcuma purpurascens* BI. commonly known as ‘Koneng Tinggang’ and ‘Temu Tis’ is a member of the Zingiberaceae family comprising 50 genera and over 1000 species, which generally consists of herbaceous plants from tropical forests [15-17]. *C. purpurascens* is an herb (55–70 cm × 19–23 cm) with branched rhizome, which grows spontaneously in teak forest. Same as other curcuma species, its rhizome is extensively used as a spice and as folk medicine [18]. Rural communities in Indonesia use this plant for treatment of wounds, boils, cough, itch, scabies and fever [16,19]. In this study, we investigated the cytotoxic effect of dichloromethane extract of *C. purpurascens* rhizome (DECPR) on human colon cancer HT-29 cells. Additionally, we also evaluated the apoptotic-inducing effect of DECPR and suggested a possible molecular mechanism underlying the observed activity.

## Methods

### Sample collection and preparation of extracts

The *C. purpurascens* rhizome studied in this work was collected from Yogyakarta, Indonesia. The botanical identification was made by Mr. Teo Leong Eng., Faculty of Science, University of Malaya. A voucher specimen (KL 5793) was deposited in the herbarium of the Chemistry department, Faculty of Science, University of Malaya, Kuala Lumpur, Malaysia. The air-dried and powdered rhizomes (1.0 kg) were subjected to extraction using *n*-hexane followed by dichloromethane. The resulting filtrate of the rhizome was concentrated by a rotary evaporator at 40°C. The extracts were stored at −20°C and dissolved in dimethyl sulfoxide (DMSO) for further *in vitro* experiments. Samples of DECPR administered to animals were dissolved in the vehicle 10% Tween 20.

### Cell lines and culture conditions

CCD841 (normal human colon epithelial cell line), HepG2 (human hepatoma cell line), HT-29 (human colon cancer cell line), MDA-MB-231 (human breast cancer cell line), PC-3 (human prostate cancer cell line) and WRL-68 (human hepatic cell line) were purchased from the American Type Culture Collection (ATCC, Manassas, VA, USA) and maintained in RPMI-1640 (Sigma, St. Louis, Mo, USA) supplemented with 10% fetal bovine serum (PAA Lab, Pashing, Austria), 100 U/mL penicillin and 100 mg/mL streptomycin (PAA Lab) in a humidified atmosphere with 5% CO_2_ at 37°C. Untreated medium containing vehicle DMSO (0.1%) was used as a negative control in this study.

### Cell proliferation assay

*In vitro* cytotoxic effect of DECPR was determined using MTT assay as previously described [20]. In brief, cells (5 × 10^4^ cells/mL) were treated with various concentrations of DECPR in a 96-well plate and incubated for 24 h. After incubation time, the MTT solution (5 mg/mL, 20 μL) was added and the plate was incubated for 2 h. DMSO (100 μL) was then used to dissolve the formazan crystals. The absorbance was measured at 570 nm with a microplate reader (Asys UVM340, Eugendorf, Austria). The suppressive effect of DECPR was expressed as IC_50_ value. The anticancer drug, 5-Fluorouracil (5-FU, Sigma, St. Louis, MO, USA) was used as a positive control towards HT-29 cells.

### GC-MS-TOF analysis of DECPR

The gas chromatography analysis of DECPR was performed using an Agilent and LECO. RESTEK, Rxi-5MS capillary column (30 m, 0.25 mm i.d., 0.25 μm film thickness) and a mass spectrometer Pegasus HT High Throughput TOFMS, as previously described in detail [21]. Compounds were identified from their mass spectra, by comparison of the retention times of peaks with interpretation of MS fragmentation patterns from the National Institute of Standards and Technology (NIST147) Mass Spectral Database.

### Acute toxicity study

Adult female *Sprague–Dawley* rats weighing 180–200 g and aged 6–8 weeks were purchased from the Animal Experimental Unit, University of Malaya, Kuala Lumpur, Malaysia. The rats were kept in cages of six animals each, with food and water ad libitum at 27 ± 2°C temperature with a 12 h light/dark cycle and with lights on at 07.00 h. The animals were maintained and used after approval by the Animal Ethics Committee, Faculty of Medicine, University of Malaya (Ethics No. 2014-03-05/PHAR/R/ER), which is based on the guide for the Care and Use of Laboratory Animals prepared by the National Academy of Sciences and published by the National Institute of Health.

Acute toxicity (if any) of DECPR was carried out as previously described in detail [22]. In brief, a total of 18 female rats were divided into 3 groups (*n* = 6) and administered orally with vehicle (10% Tween 20, 5 mL/kg), DECPR (low dose, 2 g/kg) and DECPR (high dose, 5 g/kg). Animals were monitored for any sign of toxicity, including clinical, toxicological symptoms and mortality over a period of 2 weeks. On day 15^th^, rats were sacrificed for further histological, hematological and serum biochemical evaluations.

### Lactate dehydrogenase (LDH) release assay

Release of LDH from treated colon cancer cells was measured using Pierce LDH Cytotoxicity Assay Kit (Thermo Scientific, Pittsburgh, PA, USA), as previously described [23]. Briefly, HT-29 cells were treated with different concentrations of DECPR for 24 h. To determine the LDH leakage, the supernatant of treated cells was transferred in 96-well plate. Triton X-100 (2%) as positive control was used to completely lyse the cells and release the maximum LDH. Then, the LDH reaction solution (100 μl) was added to the cells for 30 min. The red color intensity representing the LDH activity was measured at the absorbance of 490 using a Tecan Infinite 200 Pro (Tecan, Männedorf, Switzerland) microplate reader. The level of released LDH from treated colon cancer cells was expressed as a percentage of positive control.

### Cytoskeletal arrangement assay

To determine the effect of DECPR on the cytoskeletal rearrangement, we used Cellomics Cytoskeletal Rearrangement Kit (Thermo Scientific, Pittsburgh, PA, USA) as previously described in detail [24]. In brief, HT-29 cells at the exponential phase of growth were treated with different concentrations of DECPR for 24 h. Then, treated cells were fixed and stained with Hoechst and phalloidin dyes according to the vendor’s instructions. At the end, the stained cells were examined for changes in cytoskeletal rearrangement using ArrayScan HCS system (Cellomics Inc, Pittsburgh, PA, USA).

### Reactive oxygen species (ROS) generation assay

To determine the effect of DECPR on ROS generation in colon cancer cells, we carried out ROS assay [25]. Briefly, HT-29 cells (1 × 10^4^ cells/well) were seeded in a 96-well plate for 24 h. Then, colon cancer cells were treated with different concentrations of DECPR and incubated overnight. After the incubation time, treated cells were stained with dihydroethidium (2.5 μg/mL, 50 μL) and Hoechst 33342 (500 nM) for 30 min. Next, cells were fixed with paraformaldehyde (3.5%) for 15 min and washed with PBS twice. Formation of ROS in treated cells was measured using a Cellomics ArrayScan HCS reader (Cellomics Inc, Pittsburgh, PA, USA).

### Multiple cytotoxicity assay

The important characterizations of mitochondrial dependent apoptosis, including changes in cytochrome *c* release and mitochondrial membrane potential (MMP) in addition to cell membrane permeability were measured using a Cellomics Multiparameter Cytotoxicity 3 Kit as previously described in details by Lövborg and colleagues [26]. The fixed and stained HT-29 cells were exhaustively analyzed using the ArrayScan HCS system (Cellomics Inc, Pittsburgh, PA, USA).

### DNA fragmentation assay

A suicide- Track DNA Ladder isolation kit (Calbiochem, San Diego, CA, USA) was used to observe DNA fragments (mononucleosome and oligonucleosomes) formed during apoptosis. In brief, HT-29 cells were seeded at the density of 5 × 10^4^ cells/mL in a culture flask, and incubated for 24 h. They were then treated with DECPR at 12.5 and 25 μg/mL concentrations for 24 h. The treated cells were trypsinized and centrifuged at 1,000 rpm for 10 min. The pellet was gently re-suspended in 55 μl of solution #1 (kit component). It was then mixed with 20 μl of re-suspension buffer. To detect the DNA ladder, the extracted DNA samples were run on the 1.5% agarose gel in a Tris-acetic- EDTA buffer. After electrophoresis, the gel was stained with Novel Juice Cat No. LD001-1000, visualized with a UV light transilluminator, and photographed.

### Caspase-3, −8 and −9 activities assay

Activation of caspases was measured using the fluorometric assay kit (Calbiochem), according to the manufacturer’s protocol. In brief, HT-29 cells at the exponential phase of growth were supplemented with DECPR at different concentrations for 24 h. The treated cells were harvested prior to the preparation of cell lysates as described previously [27]. After the incubation, the cell lysates were treated with caspase inhibitors (caspase-3: DEVD-CHO; caspase-8: z-IETD-FMK; caspase-9: z-LEHD-FMK). After 30 min, reaction buffer and fluorogenic peptide substrate (10 μL): Ac-DEVD-AMC (caspase-3), Ac-IETD-AMC (caspase-8) and Ac-LEHD-AMC (caspase-9) were added to the cell lysates, and incubated at 37°C in the dark for 2 h. The activation of caspases in DECPR-treated HT-29 cells was measured using the Tecan Infinite 200 Pro (Tecan, Mannedorf, Switzerland) microplate reader at excitation of 390 nm and emission of 500 nm [24].

### Quantitative PCR analysis for Bax, Bcl-2 and Bcl-xl

The gene expression of Bax, Bcl-xl and Bcl-2 proteins in treated HT-29 cells was measured by quantitative PCR analysis [24]. Briefly, Zymo Research Quick-RNA MiniPrep kit (Zymo Research, Freiburg, Germany) was used to isolate total RNAs of HT-29 cells after 24 h treatment with DECPR at IC_50_ concentration. Next, complementary DNAs was synthesized using High Capacity RNA-to-cDNA kit (Applied Biosystems, Foster City, CA, USA), according to the vendor’s protocol. Quantitative PCR was performed with TaqMan Fast Advanced Master Mix and TaqMan Gene Expression Assays using the Applied Biosystems StepOnePlus Real-Time PCR (Applied Biosystems, Foster City, CA, USA). The IDs for TaqMan Gene Expression Assays used in this study are Bax: Hs00180269_m1, Bcl-xl: Hs00236329_m1, Bcl-2: Hs00608023_m1 and GAPDH: Hs02758991_g1.

### Western blotting

To confirm the changes of mRNA expression detected from quantitative PCR analysis, western blot assay was performed as previously described [24]. In brief, after treatment of HT-29 cells (1 × 10^6^ cells/mL) with DECPR at different concentrations for 24 h, cells were washed with PBS and lysed in ice-cold Radio Immuno Precipitation Assay buffer (RIPA, Thermo Scientific (Rockford, IL, USA). Protein separation was carried out on 10% resolving polyacrylamide gels (i.e., SDS-PAGE) followed by electroblotting at 25 mA for 2 h. After transforming the proteins to polyvinylidene fluoride membrane (Pierce, Rockford, IL, USA), the membrane was blocked using a Blocker Casein (Pierce). The blots were incubated overnight with specific primary antibodies; β-actin 1:10000 (Cat: ab6276, Abcam), Bax 1:1000 (Cat: ab7977, Abcam), Bcl-2 (1:1000 Cat: ab18210, Abcam), Bcl-xl (1:1000 Cat: ab32310, Abcam), and then exposed to peroxidase-coupled secondary antibodies for 2 h. Subsequent detection of protein expression was carried out using the Fusion FX7 system (Vilber Lourmat, Eberhardzell, Germany).

### Statistical analysis

The results were presented as the mean ± SEM of the three independent experiments. A one-way analysis of variance (ANOVA) was performed using the prism statistical package (GraphPad Software, USA). *P*< 0.05 was considered statistically significant.

## Results

### DECPR showed the strongest cytotoxic effect on HT-29 cells

To determine the effects of hexane and dichloromethane extracts of *C. purpurascens* rhizome on human cancer cells, the viability of four different cancer cell lines after 24 h treatment with the extract was examined using MTT assay. As shown in Table 1, hexane and dichloromethane extracts showed the extensive range of cytotoxic effects towards cancer cells (IC_50_ ranged from 7.79 ± 0.54 to 31.10 ± 1.45 μg/mL). The results showed that DECPR induced the higher cytotoxic effects on cancer cells when compared with the hexane extract. Furthermore, DECPR elicited the strongest anti-proliferative effect on HT-29 colon cancer cells with the IC_50_ value of 7.79 ± 0.54 μg/mL. 5-FU, the standard anticancer drug, exhibited the IC_50_ value of 4.56 ± 0.67 μg/mL against HT-29 cells after 24 h treatment. The relatively higher IC_50_ value of DECPR on WRL-68 and CCD841 compared to the cancer cells suggested the safety of this plant on normal human cells and its selective cytotoxic effects. Since DECPR showed the strongest suppressive effect towards HT-29 cells, we carried out the further experiments only on the respective colon cancer cell.

**Table 1:** **IC**_**50**_ **values of** ***C. purpurascens*** **rhizome extracts on six different cell lines after 24 h treatment**

**Extract**	**Cell lines, IC**_**50**_ **(μg/mL)**					
	**HT-29**	**HepG2**	**PC-3**	**MDA-MB-231**	**CCD841**	**WRL-68**
Hexane	14.16 ± 1.04	16.78 ± 1.45	18.21 ± 0.91	31.10 ± 1.45	61.28 ± 2.41	73.27 ± 3.41
Dichloromethane	7.79 ± 0.54	10.26 ± 0.78	12.41 ± 1.01	18.48 ± 1.65	57.46 ± 2.34	69.74 ± 3.85

The data represent the means ± SEM of three independent experiments.

### GC-MS-TOF profile of DECPR

**Figure 1:**
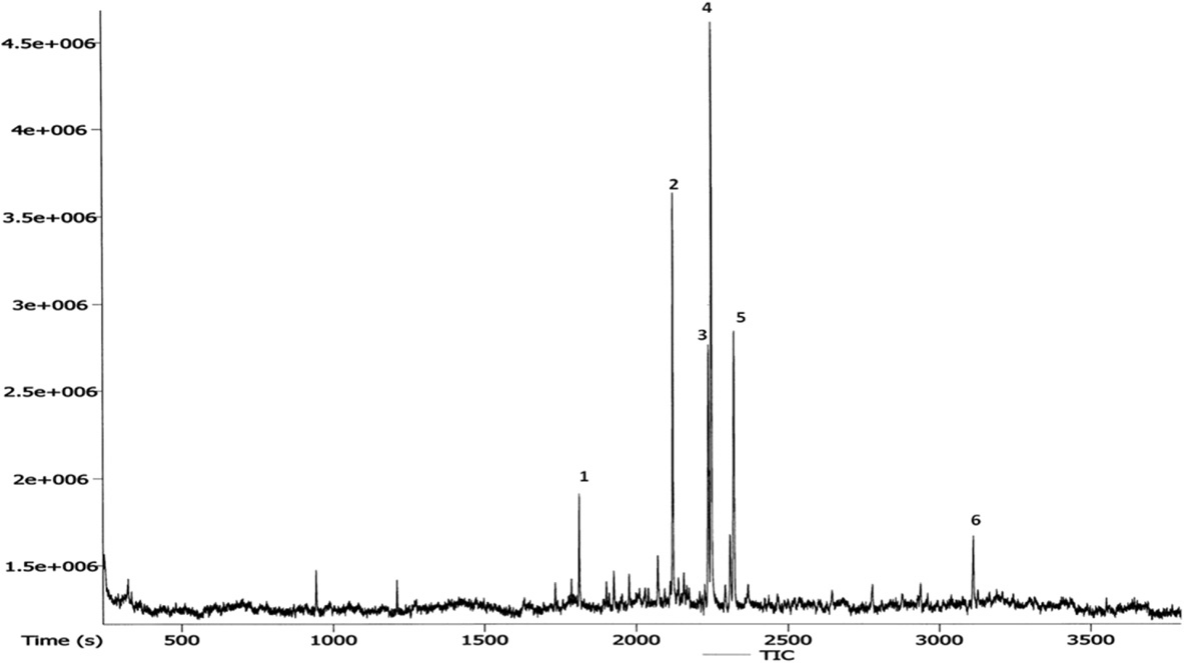
**A GC-MS-TOF chromatogram of the chemical constituents of DECPR.**

As shown in Figure 1, the dichloromethane extract of *C. purpurascens* was characterized with a GC-MS-TOF analysis. The gas chromatography profile of DECPR showed that the detected compounds in DECPR are c-Elemene (1), a-elemenone (2), ar-turmerone (3), turmerone (4) and curlone (5), with turmerone (4) as the major compound (Table 2).

**Table 2:** **The possible compounds in DECPR were characterized using a GC-MS-TOF analysis**

**Peak no.**	**Name of compounds**	**Retention time (s)**	**Mass**
1	c-Elemene	1810.45	189
2	a-Elemenone	2119	218
3	ar-Turmerone	2235.55	216
4	Turmerone	2245.75	218
5	Curlone	2319.85	218

### Safety of DECPR

**Figure 2:**
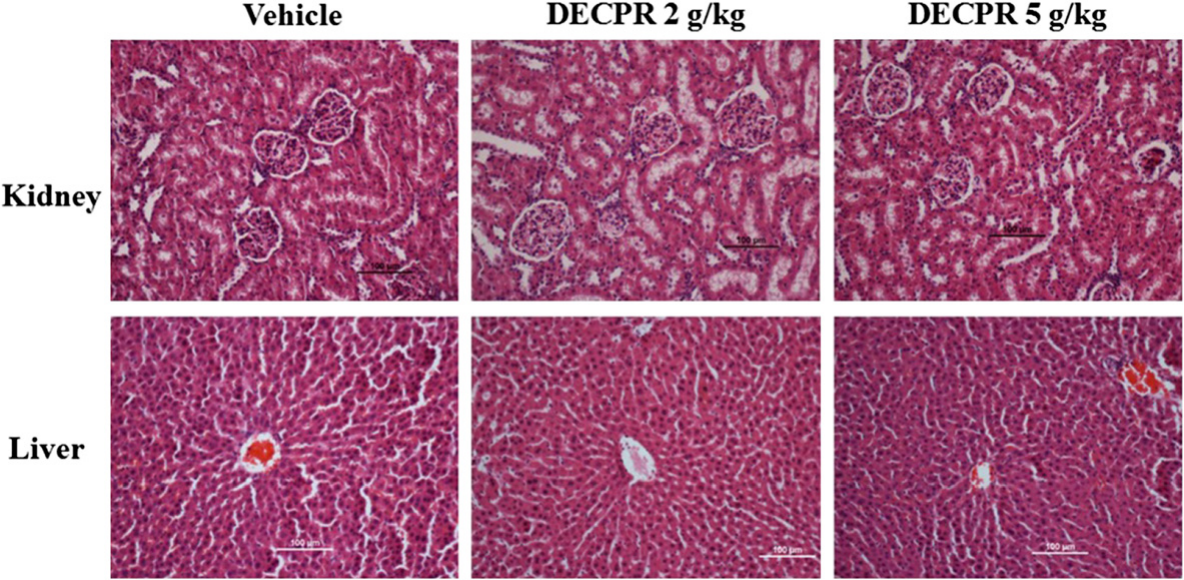
**Histological analysis.** Histological sections of kidney and liver in rats after treatment with 5 mL/kg vehicle (10% Tween 20), DECPR (2 g/kg) and DECPR (5 g/kg) showed no sign toxicity in these organs.

In the acute toxicity analysis, the rats were orally administered with DECPR at dosages of 2 g/kg and 5 g/kg and were monitored for 2 weeks. The result of study did not reveal any report for mortality or any sign of toxicity in rats. There was no detectable sign of toxicity in serum biochemical and hematological parameters of the treated groups compared with the vehicle group (Additional file 1: Table S1). The histological analysis showed the structures of the kidney and liver remain normal confirming the safety of DECPR at the doses tested (Figure 2).

### Induction of LDH leakage by DECPR

**Figure 3:**
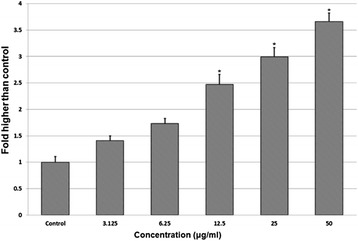
**LDH assay elicited the cytotoxicity of DECPR towards HT-29 cells.** The result showed significant LDH release at concentrations of 12.5 to 50 μg/mL, in a dose-dependent manner. The data represent the means ± SEM of three independent experiments. *P < 0.05 compared with the untreated group.

Any kind of cell’s demise, including apoptosis and necrosis is associated with LDH release caused by irreversible cell membrane damage. Hence, measurement of the LDH leakage from cells into the medium is an easy marker to determine the cytotoxic activity [28]. In order to confirm the anti-proliferative effect of DECPR observed in MTT assay, we measured the level of released LDH from HT-29 cells after 24 h of treatment. As depicted in Figure 3, DECPR caused a significant and dose-dependent elevation in LDH release at 12.5 to 50 μg/mL concentrations, compared to the control. The results of MTT and LDH assays together confirmed the noticeable antiproliferative effect of DECPR towards HT-29 cells.

### Induction of cytoskeletal rearrangement and nuclear fragmentation by DECPR

**Figure 4:**
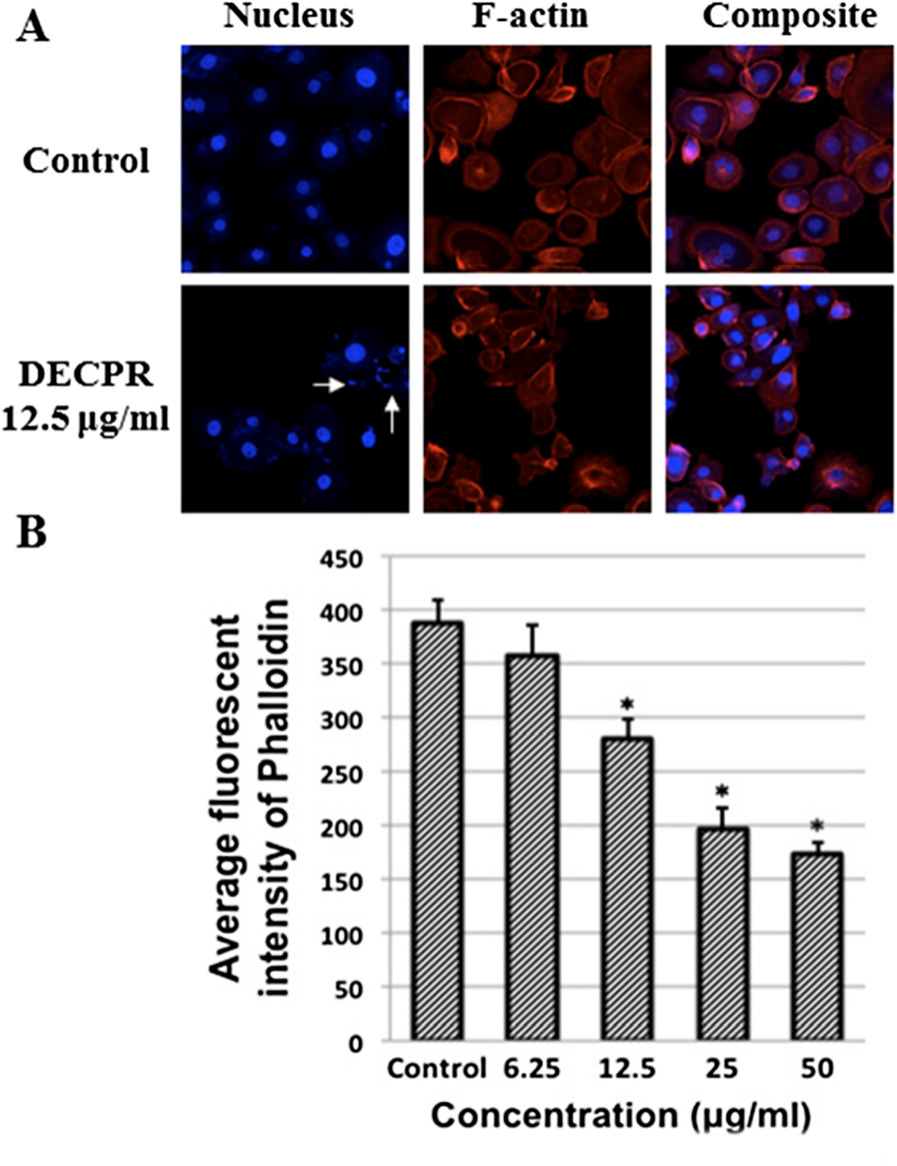
**Effect of DECPR on cell membrane permeability, MMP and cytochrome c release. (A)** HT-29 cells were treated with different concentrations of DECPR for 24 h and stained with Hoechst 33342 (blue) for nucleus and phalloidin (red) for polymerized actin (F-actin). White arrows depict DNA fragmentation and shrinkage. **(B)** Representative bar chart showed the significant reduction in fluorescent intensity of phalloidin at 12.5 to 50 μg/mL concentrations showing the cytoskeletal rearrangement induced by DECPR. The data represent the means ± SEM of three independent experiments. *P < 0.05 compared with the untreated group.

Since DECPR showed a significant cytotoxic potential towards HT-29 cells, further mechanistic studies were carried out with this extract. To investigate the cytoskeletal and nuclear changes in HT-29 cells after DECPR treatment, Hoechst 33342 and phalloidin dyes were used to detect perturbation in nucleus and F-actin, respectively. As illustrated in Figure 4A, HT-29 treated cells elicited the clear sign of DNA shrinkage and fragmentation after 24 h of treatment. In addition, F-actin perturbations at the peripheral membrane of HT-29 cells revealed the cytoskeletal rearrangements induced by DECPR, which showed to be significant at 12.5 to 50 μg/mL concentrations (Figure 4B). These morphological changes suggest the induction of apoptosis by DECPR.

### Induction of ROS generation by DECPR

**Figure 5:**
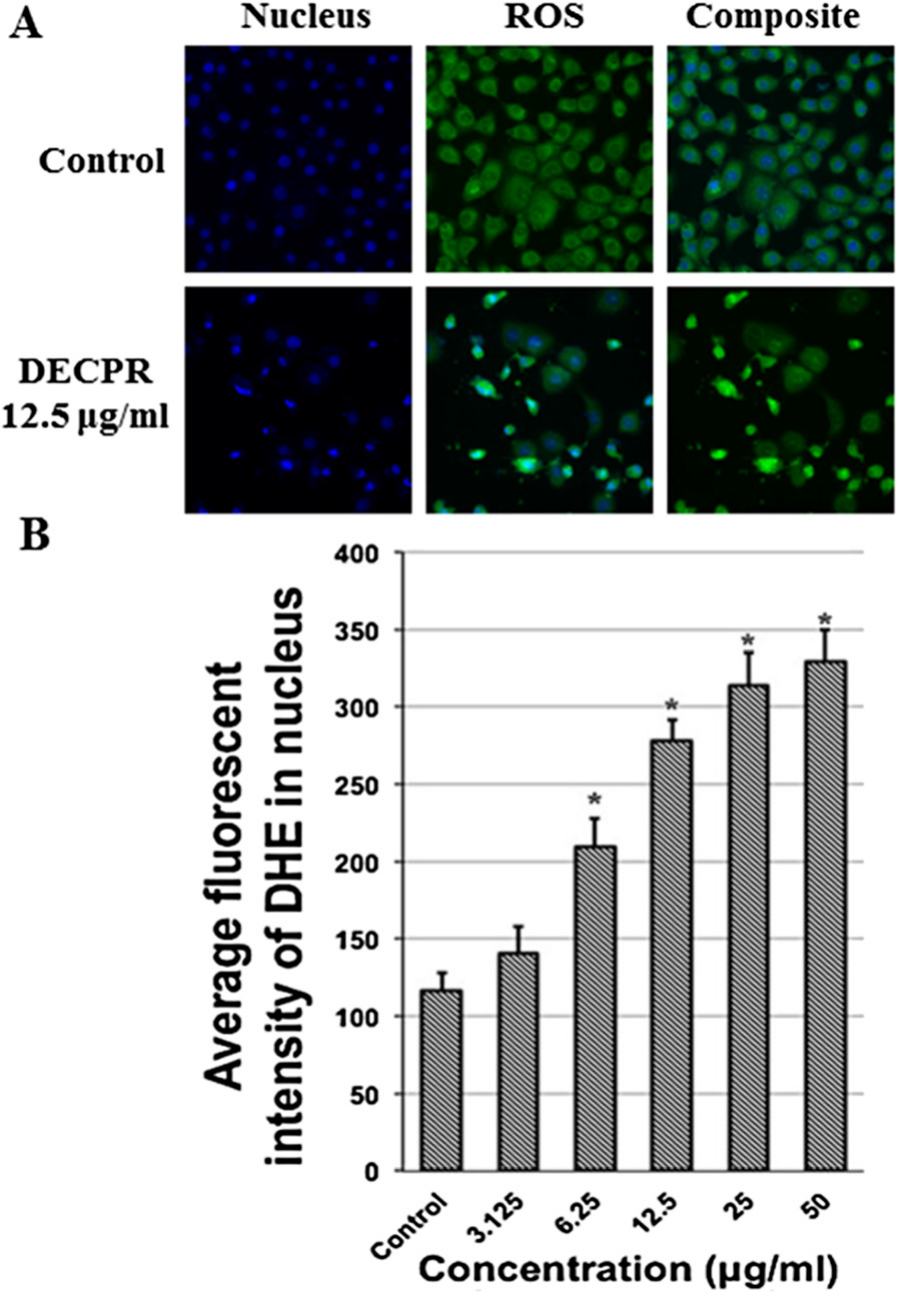
**Effect of DECPR on ROS generation in HT-29 cells. (A)** HT-29 cells were treated with vehicle DMSO or DECPR at 12.5 μg/mL concentration, stained with Hoechst 33342 dye (blue) for nucleus and dihydroethidium for ROS detection. **(B)** Representative bar chart showed significant ROS generation at 6.25 to 50 μg/mL concentrations after 24 h treatment with DECPR. The data represent the means ± SEM of three independent experiments. *P < 0.05 compared with the untreated group.

The level of ROS in HT-29 cells after DECPR treatment was examined using the oxidative-sensitive dihydroethidium probe which converts to fluorescent ethidium and intercalates into DNA as a result of ROS generation. When HT-29 cells were treated with DECPR for 24 h, ethidium derived fluorescence dose-dependently increased showing the capacity of DECPR to cause intracellular oxidation (Figure 5A). Quantification of the generated fluorescent intensity with HSC system demonstrated significant ROS production at 6.25 to 50 μg/mL concentrations (Figure 5B). The level of ROS was elevated to more than 2-fold at 12.5 μg/mL concentration compared to the control. This result showed that DECPR can promote oxidative stress in HT-29 cells upon treatment.

### Induction of mitochondrial-initiated events by DECPR

**Figure 6:**
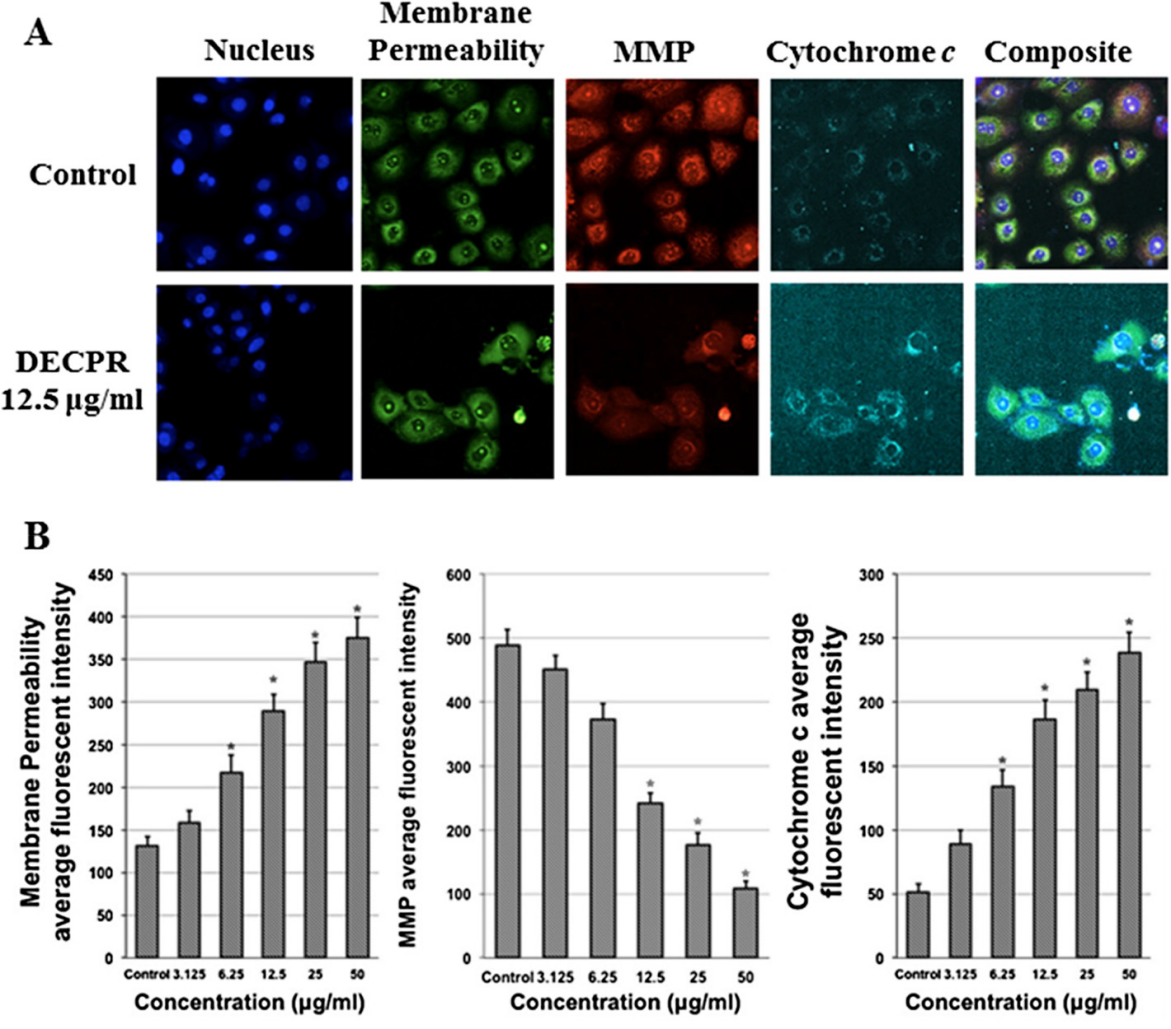
**Simultaneous analysis of DECPR effect on three different apoptotic markers. (A)** HT-29 cells were treated with DECPR at different concentrations for 24 h. After fixing the cells, they were stained with Hoechst 33342, membrane permeability, MMP and cytochrome *c* dyes following with HCS analysis of the stained cells. **(B)** Representative analysis of the figures showed that DECPR at 6.25 to 50 μg/mL concentrations significantly elevated the cell membrane permeability and cytochrome *c* release. Meanwhile, at 12.5 to 50 μg/mL concentrations, there was a significant collapse in MMP. The data represent the means ± SEM of three independent experiments. *P < 0.05 compared with the untreated group.

To determine the possible mechanism of action in which DECPR suppressed the proliferation of HT-29 cells, we investigated the changes in the three critical apoptosis-related factors, namely cell membrane permeability, MMP and cytochrome *c*. As depicted in Figure 6A, cell membrane permeability in control cells was marked with green fluorescent dye, which was conspicuously elevated in DECPR-treated cells. Meanwhile, the red fluorescent intensity of control cells was noticeably attenuated after DECPR treatment showing the collapse in MMP (Figure 6A). In addition, the cyan fluorescent intensity in control cells presenting the cytochrome *c* leakage from mitochondria to cytosol was markedly lightened upon treatment with DECPR (Figure 6B). The quantitative analysis of the fluorescent intensity of three markers showed a dose-dependent and significant increase in cell membrane permeability and cytochrome *c* leakage at 6.25 to 50 μg/mL concentrations. The MMP of treated HT-29 cells was also significantly reduced at these concentrations.

### Induction of DNA fragmentation by DECPR

**Figure 7:**
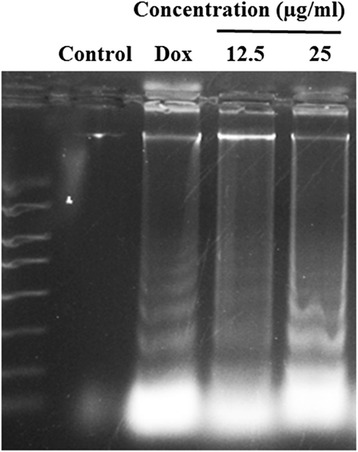
**DECPR induced DNA fragmentation in HT-29 cells.** Cells were exposed to DECPR at 12.5 and 25 μg/mL concentrations for 24 h. Doxorubicin (Dox, 10 μg/mL) was used as a positive control. After incubation, the extracted DNA samples were run on the 1.5% agarose gel in a Tris-acetic- EDTA buffer, stained and visualized with a UV light transilluminator.

To elucidate late stage of apoptosis, DNA laddering assay was performed. The exposure of HT-29 cells to DECPR at 12.5 and 25 μg/mL concentrations for 24 h led to apparent DNA fragmentation, as shown by the formation of DNA ladders in the agarose gel (Figure 7), whereas the control did not reveal any DNA fragmentation. The ability to cause DNA fragmentation is one of the hallmarks of late apoptotic cell death.

### Induction of caspases activation by DECPR

**Figure 8:**
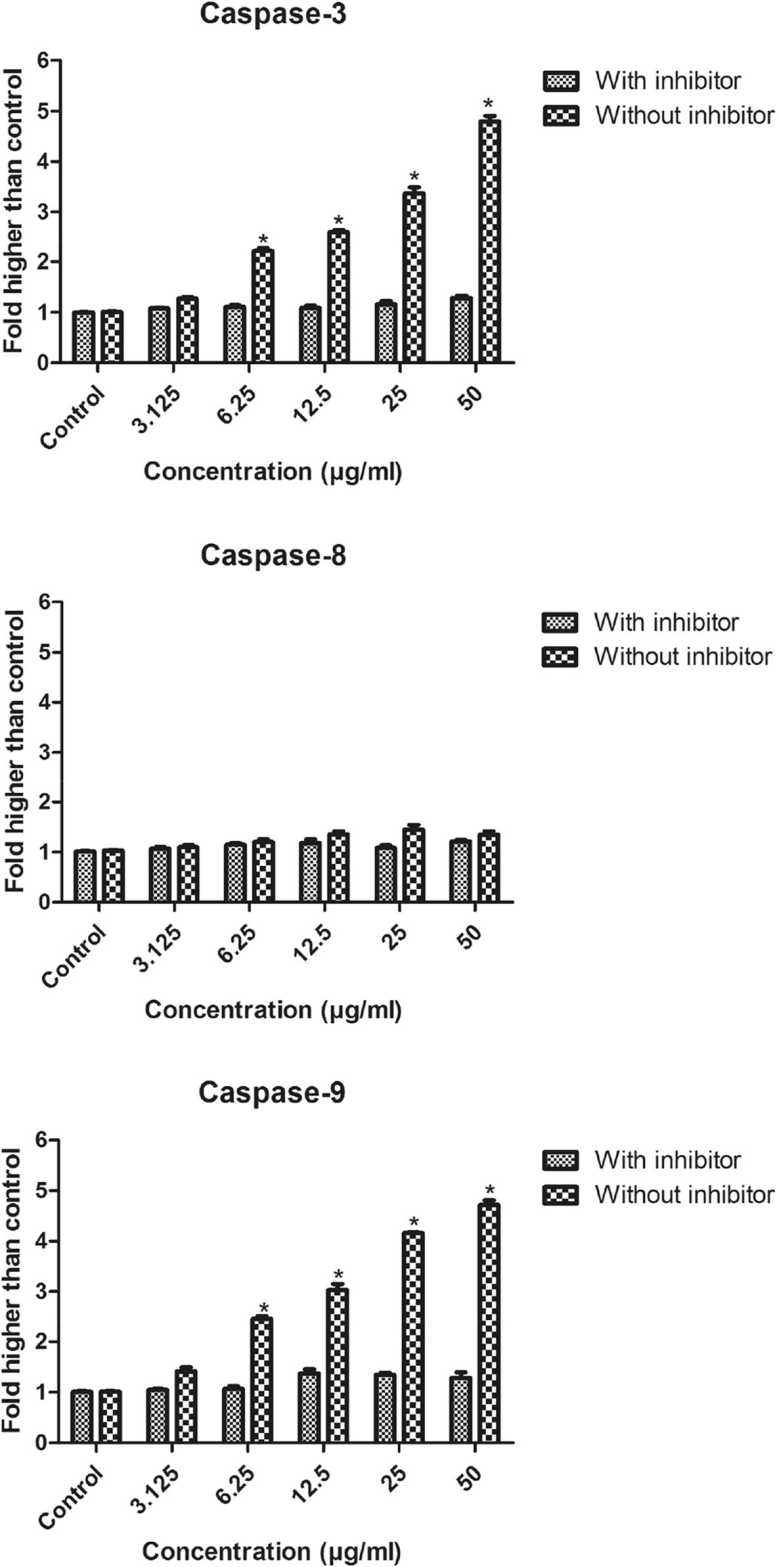
**DECPR induced caspase-3 and −9 activation in HT-29 cells.** Cells were treated with different concentrations of DECPR for 24 h, in the presence or absence of specific inhibitors, following with a fluorometric analysis of caspases activities. Treated cells without inhibitors showed the significant activation for caspase-3 and −9 at 6.25 to 50 μg/mL concentrations, while there was no significant fold-change in caspase-8 activity. The data represent the means ± SEM of three independent experiments. *P < 0.05 compared with the untreated group.

Since morphological and biochemical characterizations of apoptosis were observed in DECPR-treated HT-29 cells, we measured the activity of different caspases as key mediators of apoptosis using a fluorometric analysis. Between initiator caspase-8 and −9, DECPR only induced the activity of caspase-9 with dose-dependent and significant elevation at 6.25 to 50 μg/mL concentrations. Meanwhile, the activity of initiator caspase-8 elicited no significant perturbation. In addition, the activation of executioner caspase-3 was significantly increased at the same concentrations (Figure 8). To prove the role of caspases in DECPR-induced apoptosis, specific inhibitors of caspases were supplemented in HT-29 treated cells. In the presence of inhibitors, fold elevation of caspase-3 and caspase-9 significantly decreased (Figure 8). The results of caspases activity associated with MMP changes and cytochrome *c* release strongly suggest the induction of mitochondrial-mediated apoptosis by DECPR.

### Changes in expression of apoptosis-associated molecules by DECPR

**Figure 9:**
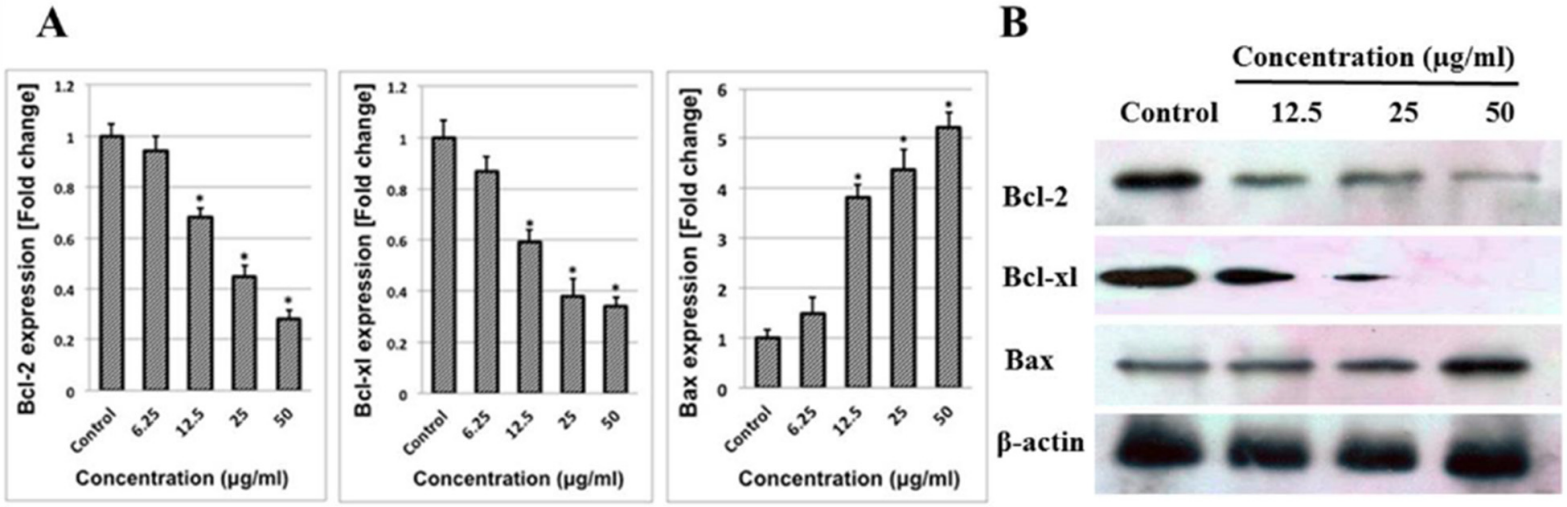
**DECPR induced changes in expression of mitochondrial-dependent proteins. (A)** Quantitative PCR analysis of apoptosis-associated genes in HT-29 cells showed significant up-regulation of Bax and down-regulation of Bcl-2 and Bcl-xl at the mRNA level. The data represent the means ± SEM of three independent experiments. *P < 0.05 compared with the untreated group. **(B)** Western blot analysis confirmed up-regulation of Bax and down-regulation of Bcl-2 and Bcl-xl at the protein level.

Bcl-2 family of proteins has critical role in the regulation of mitochondrial-mediated apoptosis, and perturbation in the expression of these genes trigger a variety of biochemical changes. Hence, we examined the mRNA expression of the pro-apoptotic and anti-apoptotic molecules upon treatment with DECPR. Pro-apoptotic gene, Bax showed dose-dependent and significant mRNA up-regulation after 24 h treatment with DECPR at 12.5 to 50 μg/mL concentrations*.* Meanwhile, at the same concentrations, there was a significant down-regulation in the mRNA expression of Bcl-2 and Bcl-xl (Figure 9A). These changes were also confirmed at the protein level using a western blot analysis (Figure 9B).

## Discussion

Plants from *Curcuma* genus represented by the turmeric spice, such as *C. longa*, *C. phaeocaulis*, *C. wenyujin* and *C. kwangsiensis* are well known to be valuable herbal medicines [29]. Due to the extensive biological activity reported for the chemical constituents of these plants, including anti-Alzheimer’s disease [30], anti-inflammatory [31], anti-angiogenesis [32] and anti-microbial [33], a number of scientific works have been carried out on *Curcuma* species. The safety of these plants and their active compounds in animal studies in a number of species have been confirmed. However, some species were found to be susceptible to hepatotoxicity at high doses of turmeric [34,35]. The present study demonstrated the safety of DECPR in rats even at high dose of 5 g/kg without any sign of renal or hepatic dysfunction. In spite of numerous studies on *Curcuma* species, there is no report on biological activity of *C. purpurascens*, in special anti-cancer potential. Hence, in the current experiment, cytotoxic effect of *C. purpurascens* on different cancer cells was examined. Our result showed a promising suppressive effect of DECPR against HT-29 cells observed by MTT and LDH assays.

The growing body of experimental evidences supports the extensive anticancer effect of *Curcuma* species and their chemical constituents, known as curcuminoids, towards different cancer cells [36-40]. *In vivo* studies and clinical trials for the treatment of various tumors have highlighted the role of curcuminoids and their analogues as promising therapeutic agents. Turmerone and its derivatives as major compounds detected in DECPR were previously reported to have apoptotic effect towards different cancer cells, such as HL-60, MCF-7, MDA-MB-231 and HepG2 cancer cells [41,42]. However, the apoptotic effect of turmerone and its mechanism of action on HT-29 cells have been not fully illustrated yet.

Apoptosis generally defines with specific morphological characterizations, including cell shrinkage, nuclear or cytoplasmic fragmentations, chromatin condensation and formation of dense bodies that are phagocyted by neighboring cells [43]. It is well established that restructuring of actin cytoskeleton has a critical role in the formation of morphological changes in apoptotic cells [44]. Our study indicated that DECPR treatment induced nuclear shrinkage and fragmentation after 24 h. This result was associated with alteration in actin filaments polymerizations in the treated HT-29 cells. Previous studies showed that standard anticancer drugs such as cisplatin and tamoxifen cause F-actin damages prior to the induction of nuclear changes in cancer cells [45,46].

ROS can generate nonradical derivatives of oxygen, such as H_2_O_2_ and free radicals, namely hydroxyl radical (OH^−^) and superoxide (O^2^) [47]. The excessive production of free radicals has been demonstrated to accelerate cell death by inducing severe damages to DNA, lipid membranes and proteins of cells [48]. In the current study, we found that DECPR significantly induced ROS generation in HT-29 cells. A previous study showed that prime initiating signaling factor of ROS was markedly involved in three different signaling pathways of apoptosis induced by curcumin in L929 cells showing the critical role of ROS generation in the suppressive effect of different anticancer agents [49]. It is well-established that numerous apoptotic stimuli, including ROS generation, initiate apoptosis by disturbing mitochondrial function sufficiently to trigger leakage of cytochrome *c* from mitochondria [50]. Production of MMP across the mitochondrial inner membrane is the result of utilization of oxidizable substrates, therefore excessive generation of ROS in cells may lead to the loss in MMP [51]. This is showed by HSC analysis on HT-29 cells after DECPR treatment which demonstrated significant reduction in MMP and elevation in cell membrane permeability and cytochrome *c* release.

Cytochrome *c* release from mitochondria to cytosol activates the initiator and executioner caspases involved in the intrinsic pathway of apoptosis [52,53]. The biochemical changes of this apoptotic pathway are mediated and tightly regulated by Bcl-2 family of proteins, including pro-apoptotic and anti-apoptotic proteins [54]. Cytochrome *c* release is mediated through dimerization of Bax and Bak as a pro-apoptotic proteins [55]. On the other hand, activity of pro-apoptotic proteins is facilitated through inhibition of anti-apoptotic proteins, e.g. Bcl-2 and Bcl-xl by intracellular stress signals [56]. In the present experiment, DECPR caused a dramatic cytochrome *c* leakage to cytosol in HT-29 cells as a key factor in apoptosome formation which leads to the cleavage of procaspase-9 to caspase-9 [57]. The investigation on the activation of caspases upon treatment with DECPR demonstrated the elevation in the activity of caspase-3 and −9. According to these data, it is strongly conceivable that ROS production in HT-29 cells triggered the mitochondrial-initiated events leading to the cytochrome *c* release and activation of caspase cascade. Our investigations on the mRNA expression of apoptosis-associated proteins also supported the induction of apoptosis through mitochondrial-mediated pathway.

## Conclusions

This is the first report on the noteworthy suppressive effect of *Curcuma purpurascens* BI. rhizome against cancer cells. Our results strongly suggest that DECPR induced apoptosis in HT-29 cells by activating the mitochondrial death pathway via the involvement of Bcl-2/Bax/Bcl-xl and ROS production. However, we do not know whether apoptosis induced by DECPR relies on a single or combined effects of different compounds detected in this extract. Therefore, further study with bioassay guided approach is still required to fully explain the apoptosis-inducing effect of DECPR and the chemical constituents involved in this activity.

## Additional file

Additional file 1: Table S1. Effects of DECPR on (A) renal and (B) liver function tests and (C) hematological parameters of rats after 2 weeks of acute toxicity study.

## Abbreviations

DECPR: Dichloromethane extract of *Curcuma purpurascens* BI. rhizome
GC-MS-TOF: Gas chromatography–mass spectrometry–time of flight
LDH: Lactate dehydrogenase
ROS: Reactive oxygen species
Bax: Bcl-2 associated X protein
Bcl-2: B-cell lymphoma protein 2
Bcl-xl: Bcl-2 related protein, long isoform
MPTP: Mitochondrial permeability transition pores
MMP: Mitochondrial membrane potential
DMSO: Dimethyl sulfoxide
MTT: (3-(4,5-dimethylthiazol-2-yl)-2,5-diphenyltetrazolium)
