# Supplementary material for: Inhibitory effect of Curcuma purpurascens BI. rhizome on HT-29 colon cancer cells through mitochondrial-dependent apoptosis pathway
Source: BMC Complement Altern Med. 2015 Feb 5;15:15. doi: 10.1186/s12906-015-0534-6 (PMC4323059; doi:10.1186/s12906-015-0534-6)
Supplement: Additional file 1: Table S1. — Effects of DECPR on (A) renal and (B) liver function tests and (C) hematological parameters of rats after 2 weeks of acute toxicity study. [file 12906_2015_534_MOESM1_ESM.docx]

**Additional file 1: Table S1. Effects of DECPR on (A) renal and (B) liver function tests and (C) hematological parameters of rats after 2 weeks of acute toxicity study.**

(A)

| Group | Sodium (mM/L) | Potassium (mM/L) | Chloride (mM/L) | CO_2_  (mM/L) | Anion (mM/L) | Urea (mM/L) | Creatinine (μM/L) |
| --- | --- | --- | --- | --- | --- | --- | --- |
| Vehicle | 138.25 ± 0.46 | 4.76 ± 0.07 | 102.67 ± 0.38 | 26.78 ± 0.53 | 13.37 ± 0.46 | 4.39 ± 0.27 | 30.68 ± 1.59 |
| 2 g/kg | 139.56 ± 0.39 | 5.05 ± 0.63 | 104.37 ± 0.46 | 28.64 ± 0.54 | 14.68 ± 0.65 | 4.85 ± 0.36 | 29.87 ± 2.24 |
| 5 g/kg | 140.87 ± 0.73 | 4.89 ± 0.035 | 103.47 ± 0.48 | 27.81 ± 0.42 | 14.78 ± 0.36 | 5.16 ± 0.29 | 31.48 ± 2.17 |

(B)

| Group | Total Protein  (g/L) | Albumin  (g/L) | Globulin  (g/L) | TB  (μmol/L) | AP  (U/L) | ALT  (U/L) | AST  (U/L) | GGT  (U/L) |
| --- | --- | --- | --- | --- | --- | --- | --- | --- |
| Vehicle | 67.52 ± 0.76 | 9.48 ± 0.21 | 51.39 ± 1.41 | 2.25 ± 0.17 | 151.64 ± 5.49 | 47.54 ± 1.27 | 170.21 ± 5.28 | 2.39 ± 0.35 |
| 2 g/kg | 65.46 ± 0.38 | 8.67 ± 0.43 | 51.14 ± 1.25 | 2.21 ± 0.15 | 152.43 ± 5.68 | 44.72 ± 1.63 | 169.57 ± 6.51 | 2.48 ± 0.49 |
| 5 g/kg | 63.78 ± 0.82 | 8.68 ± 0.29 | 50.32 ± 1.27 | 2.23 ± 0.16 | 154.56 ± 6.75 | 43.69 ± 1.35 | 171.35 ± 6.49 | 2.65 ± 0.37 |

(C)

| Group | HGB  (g/dL) | HCT  (%) | RBC  (10^6^ cells/µL) | MCV  (fL) | MCH  (pg) | MCHC  (g/dL) | RDW  (%) | WBC  (10^3^cells/µL) | Platelet  (10^3^ cells/µL) |
| --- | --- | --- | --- | --- | --- | --- | --- | --- | --- |
| Vehicle | 15.42 ± 0.13 | 45 ± 0.00 | 10.56 ± 0.14 | 61.23 ± 0.59 | 17.31 ± 0.27 | 34.48 ± 0.16 | 18.12 ± 0.47 | 7.21 ± 0.44 | 988.71 ± 23.58 |
| 2 g/kg | 15.23 ± 0.12 | 44 ± 0.00 | 10.63 ± 0.17 | 62.65 ± 0.48 | 17.79 ± 0.27 | 34.45 ± 0.33 | 19.25 ± 0.61 | 7.26 ± 0.36 | 996.41 ± 25.34 |
| 5 g/kg | 15.27 ± 0.11 | 44 ± 0.00 | 10.79 ± 0.15 | 61.84 ± 0.39 | 18.62 ± 0.31 | 34.14 ± 0.28 | 20.79 ± 0.36 | 7.33 ± 0.38 | 1012.64 ± 23.21 |

Values expressed as mean ± SEM. There are no significant differences between groups. Significant value at *p*<0.05. TB: Total Bilirubin; AP: Alkaline Phosphatase; ALT: Alanine Aminotransferase; AST: Aspartate Aminotransferase; GGT: G-Glutamyl Transferase; HGB: Haemoglobin; HCT: Haematocrit; RBC: Red Cell Count; MCV: Mean Corpuscular Volume; MCH: Mean Corpuscular Haemoglobin; MCHC: Mean Corpuscular Haemoglobin Concentration; RDW, Red Cell Distribution Width; WBC, White Cell.
